# Effectiveness of participatory video in lowering stigma against people with mental, neurological and substance use disorders in Kenya

**DOI:** 10.1192/bjo.2023.587

**Published:** 2023-11-13

**Authors:** Mary A. Bitta, Judy Baariu, Simone Grassi, Symon M. Kariuki, Belinda Lennox, Charles R. J. C. Newton

**Affiliations:** Clinical Research-Neurosciences, KEMRI/Wellcome Trust Research Program, Centre for Geographic Medicine Research (Coast), Kilifi, Kenya; and Department of Psychiatry, University of Oxford, Oxford, UK; Clinical Research-Neurosciences, KEMRI/Wellcome Trust Research Program, Centre for Geographic Medicine Research (Coast), Kilifi, Kenya; Documentary Institute of Eastern Africa, Nairobi, Kenya; Clinical Research-Neurosciences, KEMRI/Wellcome Trust Research Program, Centre for Geographic Medicine Research (Coast), Kilifi, Kenya; Department of Psychiatry, University of Oxford, Oxford, UK; and Department of Public Health, Pwani University, Kilifi, Kenya; Department of Psychiatry, University of Oxford, Oxford, UK; Department of Public Health, Pwani University, Kilifi, Kenya

**Keywords:** Stigma, mental disorders, low- and middle-income country, participatory video, digital interventions

## Abstract

**Background:**

Globally, stigma associated with mental, neurological and substance use (MNS) disorders is rampant and a barrier to good health and overall well-being of people with these conditions. Person-centred digital approaches such as participatory video may reduce stigma, but evidence on their effectiveness in Africa is absent.

**Aims:**

To evaluate the effectiveness of participatory video in reducing mental health-related stigma in a resource-limited setting.

**Method:**

We evaluated the effectiveness of using participatory video and face-to-face interaction between people with MNS disorders and a target audience in lowering stigma among 420 people living in Kilifi, Kenya. Changes in knowledge, attitudes and behaviour (KAB) were measured by comparing baseline scores with scores immediately after watching the participatory videos and 4 months after the intervention. Sociodemographic correlates of stigma scores were examined using multivariable linear regression models.

**Results:**

Compared with baseline, KAB scores significantly improved at both time points, suggesting reduced stigma levels. At 4 months, the changes in scores were: knowledge (β = 0.20, 95% CI 0.16–0.25; *P* < 0.01), liberal attitude (β = 1.08, 95% CI 0.98–1.17; *P* < 0.01), sympathetic attitude (β = 0.52, 95% CI 0.42–0.62; *P* < 0.01), tolerant attitude (β = 0.72, 95% CI 0.61–0.83; *P* < 0.01) and behaviour (β = 0.37, 95% CI 0.31–0.43; *P* < 0.01). Sociodemographic variables were significantly correlated with KAB scores; the correlations were not consistent across the domains.

**Conclusions:**

Participatory video is a feasible and effective strategy in improving knowledge, attitudes and intended behaviour in a resource-limited setting. Further studies are required to understand the mechanisms through which it lowers stigma and to examine long-term sustainability and the effectiveness of multicomponent interventions.

Globally, stigma associated with mental, neurological and substance use disorders remains rampant and continues to have an impact on the health and quality of life of people with these disorders.^1–3^ Reduction of stigma in mental illness was a key priority in the World Health Organization's 2013–2020 Mental Health Action Plan. Evidence on implementation and effectiveness of stigma reduction campaigns in low- and middle-income countries (LMICs), however, is poor and, until recently,^4^ was absent for regions such as Africa.^5^ Evidence is urgently needed in LMICs, which bear a disproportionately higher burden of these disorders^6^ and where outcomes are worse owing to additional factors such as under-equipped mental health systems.^7^

The few anti-stigma interventions for mental illness conducted in LMICs have either focused on educational approaches only or have augmented educational approaches with video or in-person contact-based approaches.^8^ However, little is published about the effectiveness of participatory approaches for stigma reduction involving people with lived experience of mental illness and the target audience in creating and disseminating anti-stigma campaigns. This gap may explain the mixed results on effectiveness of these campaigns^9^ and the resulting low impact on indicators of well-being such as improved socioeconomic positions.

The use of participatory digital methods to implement stigma-reduction interventions in mental illness is gaining momentum among health advocates in both low-income and high-income settings. For example, ‘photovoice’, a method that uses photography to address challenges facing marginalised people, has been used in LMICs to address the impact of climate change on mental health.^10^ Use of films in a method called participatory video is effective in improving the understanding of mental illnesses.^11^ In participatory video, a group of marginalised people come together to script, film and produce contextually relevant educational videos about an issue that affects them. The workgroup has total control over the entire editorial process and a facilitator only guides the team on technical aspects, such as use of equipment or production software, but not on the theme and content of the films.^12^ For people with mental illness, taking part in participatory videos creates opportunities to share their experience and address stereotypes such as narratives that link mental illness with violence and crime. They also provide opportunities for changing the narrative to more positive experiences such as journeys to recovery, as was the case in one recent participatory video project.^13^

Digital interventions to reduce stigma surrounding mental illness have been successfully implemented in LMICs^4^ and the use of digital means of communication has grown exponentially globally. Participatory video has been successfully used in Kilifi, Kenya to engage communities about health research^14^ but not as a stigma reduction strategy. Our present study had two aims: (a) to evaluate the effectiveness of a participatory video project in lowering stigma against people with mental, neurological and substance use disorders; and (b) to examine the sociodemographic correlates of stigma in Kilifi, Kenya. We used knowledge, attitudes and behaviour to conceptualise stigma and used validated tools^15^ to measure the impact of the participatory video intervention on stigma scores immediately after the video exercises and 4 months later.

## Method

### Design

Between August 2020 and February 2022, we used a repeated measures design to measure the effectiveness of participatory video in lowering mental illness-related stigma by comparing baseline scores and scores at two follow-up time points: one immediately after the intervention, that is approximately 2 h after the baseline scores, and the second 4 months after the intervention. These time points are referred to here as baseline, time point 2 and time point 3 respectively. The study population were adults >18 years from a general population in Kilifi, Kenya. Written informed consent was obtained from all participants.

This was part of a larger mental illness stigma reduction campaign dubbed *Difu Simo* (‘Breaking Free’), which was established in Kilifi County in 2019 (difusimo.org/#home). *Difu Simo* conducted mass awareness campaigns in the community within Kilifi. The campaigns involved personal stories from people with lived experience of mental illness, dialogue sessions between traditional healers and biomedical practitioners, question-and-answer sessions from the audience and performances of songs, poems and dances which had messages about mental health and mental illness. Therefore, some members of the target audience for the participatory video intervention presented in this study may have participated in these mass campaigns before, during or after the participatory video intervention.

### Ethical approval

The authors assert that all procedures contributing to this work comply with the ethical standards of the relevant national and institutional committees on human experimentation and with the Helsinki Declaration of 1975, as revised in 2008. All procedures involving human subjects/patients were approved by the Scientific and Ethics Review Unit under protocol KEMRI/SERU/CGMR-C/167/3933.

### Study setting

This study was conducted in Kilifi County, which is located along Kenya's coast of the Indian Ocean. Kilifi covers an area of approximately 12 246 km^2^ and has a population of approximately 1.5 million, with 54% of the population aged between 15 and 64 years. Most of the county is rural (74%) and the main economic activities are agriculture, fishing, tourism and small-scale trade. Christianity is the most common religion (68%) and Kiswahili is Kenya's *lingua franca*.^16^ The burden of mental, neurological and substance use disorders such as depression,^17^ epilepsy,^18^ suicide^19^ and emotional and behavioural problems is high in Kilifi^20^ and people with such disorders experience stigma^21^ which is linked to cultural explanations of the causes of these disorders, such as witchcraft and curses.^22^ The mental health infrastructure and human resources are limited,^23^ with only three psychiatric out-patient units, all based in large general hospitals in large town centres.

### The intervention

The intervention was a combination of a participatory educational material (participatory videos) and a face-to-face contact-based approach. The participatory videos were created and disseminated by people with lived experience of mental illness, who are referred to here as content creators. The target audience were influential stakeholders who were familiar with the community's social structure and who were likely to initiate change in the community. They included people with lived experience of mental illness either as patients or caregivers, village elders, religious leaders, youth representatives and healthcare workers.

The study team included a project coordinator with postgraduate training in health research and two field assistants with tertiary level training in community development and mass media. Content creators were identified through the psychiatric out-patient units. Before recruitment, members of the study team gave a general talk about the study's objectives to all patients attending the psychiatric out-patient units. Those who were interested in the study were then given additional information and were invited to attend study-specific sensitisation meetings. A final list of participants who would be available for the entire duration of the study was derived and these participants were trained in the participatory video techniques.

The target audience was identified either through the County's administrative units or through snowballing. Training in the process of participatory video was conducted by InsightShare (insightshare.org/services) and all content creators plus all study team members were trained. Training on the use of filming equipment was provided by the Documentary Institute of Eastern Africa (diea.info). In total, six participatory videos focusing on schizophrenia, suicide attempts, bipolar mood disorder, substance use disorder, epilepsy and depression were created, and 420 participants (target audience) were included in the study over 55 group sessions.

Content creators chose various filming venues within Kilifi and all dissemination sessions were conducted at the Kenya Medical Research Institute's Wellcome Trust premises. This dissemination venue was selected because it was centrally located relative to the target audience and it offered amenities such as electricity, running water, security of filming equipment and strict control of COVID-19 prevention measures. The COVID-19 measures included maintaining physical distance of at least 1.5 m between individuals, use of masks and having up-to-date vaccination records. During each dissemination session, at least one content creator was present and they moderated the sessions with a member of the study team. The content creator led the film viewing and question-and-answer sessions and the study team members administered the stigma questionnaires and set up the equipment before the viewing sessions started.

### Measures

Baseline data on the following sociodemographic variables were collected: age, gender, occupation, religious affiliation, level of education, marital status, house tenure (rental or owner), experience with mental illness and exposure to other project activities before participating in the participatory video exercises.

To measure stigma, we evaluated mental health-related knowledge using the Mental Health Knowledge Schedule (MAKS),^24^ attitudes using the Community Attitudes to Mental Illness (CAMI) scale^25^ and behaviour towards people with mental illness using the Reported and Intended Behaviours Scale (RIBS).^26^ All tools were translated to Kiswahili, back-translated into English and were validated for Kilifi populations and found to possess good psychometric properties.^15^ The MAKS contains 12 questions: 6 measure general mental health knowledge and 6 measure knowledge about specific mental illnesses. The adapted and validated version of the CAMI used in this analysis contains 23 questions which measure 3 domains: authoritarianism, tolerance and sympathy. Tolerance and sympathy are positive traits, whereas authoritarianism is a negative trait which reinforces isolation of people with mental illness. Therefore, for ease of interpretation, we reverse scored the total scores in the authoritarianism domain and reported the domain as ‘liberal attitudes’.

The RIBS contains eight questions: four measure reported behaviours and four measure intended behaviours. Reported behaviours are calculated only as frequencies and are not used to calculate overall scores, hence only intended behaviours are included in the final scores. Responses for all tools are on a Likert scale and higher scores endorse positive traits, for instance a high score on the RIBS indicates greater likelihood of positive intended behaviour towards people with mental illness, whereas a low score indicates greater likelihood of stigmatising behaviour. Overall scores are calculated by summing total scores for all questions. Domain-specific scores are calculated by summing the scores from questions related to that domain.

### Statistical analysis

Descriptive participant sociodemographic statistics were presented for baseline and for time points 2 and 3, respectively. Assuming heterogeneous variance,^27^ mixed linear models were used to compare standardised scores for knowledge, attitudes and behaviour at time points 2 and 3. In all the models, time was included as a fixed effect using a categorical dummy variable. All measured sociodemographic variables were included as random covariates in the models to control for differences in distribution in the study sample. Results were reported as standard deviation units. Using a two-sided test, and assuming a standard deviation of the difference between means to be 2, the mean difference to be 0 under the null hypothesis, a correlation of 0.9 between the paired observations and a 5% significance level, a sample size of 420 was required to detect a difference in means with 100% power, after accounting for 20% attrition.

To determine whether the associations between baseline characteristics and stigma outcomes were affecting the effectiveness of the intervention, *post hoc* analyses were conducted to explore interactions between the intervention effectiveness and baseline characteristics. A negative coefficient indicated reduced stigma scores compared with baseline. For the time variable, the assumption was that there was no departure from a linear trend. All analyses were conducted in the statistical software STATA version 17 for Microsoft Windows 11.

## Results

### Sociodemographic characteristics of study participants

In total, 420 participants were recruited. All 420 completed follow-up at time point 2 and 399 (95%) completed follow-up at time point 3. There was no difference in the gender distribution of participants (*P* = 0.567) at baseline and time point 3. Participants at baseline had a median age of 38 years (interquartile range IQR = 27–51 years). There was no difference in the mean ages at baseline and at time point 3 (*P* = 0.554). The majority (92.8%) of the participants had completed at least primary level education, which is equivalent to 8 years of formal education. At baseline 69% of participants were either married or had been previously married; the rest (30.5%) were single. There were no significant differences between baseline and time point 3 distributions of levels of education, marital status, religious affiliation or occupation (Table 1).

**Table 1:** Sociodemographic characteristics of study participants

Variable	Baseline (*n* = 420)		Time 2 (post-intervention) (*n* = 420)		Time 3 (4-month follow-up) (*n* = 399)		*P* for test for difference in proportions
Age, years: median (interquartile range)	38 (26–50.5)		38 (26–50.5)		38 (27–51)		
	*n*	%	*n*	%	*n*	%	
Gender							
Male	213	50.7	213	50.7	199	49.9	0.871
Female	207	49.3	207	49.3	200	50.1	0.872
Education							
None	30	7.14	30	7.14	29	7.27	0.985
Primary	167	39.76	167	39.76	160	40.10	0.950
Secondary	164	39.05	164	39.05	155	38.85	0.971
Tertiary	59	14.05	59	14.05	55	13.78	0.967
Marital status							
Single	128	30.48	128	30.48	118	29.57	0.876
Married	240	57.14	240	57.14	231	57.89	0.869
Divorced/widowed/separated	52	12.38	52	12.38	50	12.53	0.982
House tenure							
Rental home	161	38.33	161	38.33	151	37.84	0.929
House owner	259	61.67	259	61.67	248	62.16	0.910
Religious affiliation							
None	11	2.62	11	2.62	10	2.51	0.987
Islam	108	25.71	108	25.71	104	26.07	0.952
Christianity	301	71.67	301	71.67	285	71.43	0.949
Occupation							
Employed	267	63.57	267	63.57	257	64.41	0.841
Unemployed/student/other	153	36.43	153	36.43	142	35.59	0.881
Experience with mental illness at baseline							
Yes	367	87.38	367	87.38	349	87.47	0.971
No	53	12.62	53	12.62	50	12.53	0.989
Additional contact within the project at baseline^a^							
Yes	298	70.95	298	70.95	289	72.53	0.671
No	122	29.05	122	29.05	110	27.57	0.803

a. These are people who may have joined the community awareness campaigns before being recruited to the study.

### Changes in levels of knowledge and its sociodemographic correlates

In the univariable analysis, general mental health-related knowledge significantly improved at both follow-up time points compared with baseline, as indicated by a positive effect size of β = 0.30 (95% CI 0.26– 0.34; *P* < 0.01) at time point 2 and β = 0.31 (95% CI 0.26–0.35; *P* < 0.01) at time point 3. Knowledge about specific mental illnesses also improved at both follow-up time points compared with baseline (Table 2). Being Christian (β = 0.17, 95% CI 0.01–0.34; *P* = 0.04), having a tertiary level of education (β = 0.15, 95% CI 0.03–0.27; *P* = 0.02) and having additional contact with the project (i.e. engaging in activities other than the participatory videos) (β = 0.08, 95% CI 0.02–0.14; *P* = 0.01) were significantly associated with higher general mental health-related knowledge scores. There were no significant associations between knowledge about specific mental illnesses and any sociodemographic variables.

**Table 2:** Baseline and post-intervention knowledge scores using the Mental Health Knowledge Schedule

Variable	Univariable analysis				Multivariable analysis			
	General mental health-related knowledge		Knowledge about specific mental illnesses		General mental health-related knowledge		Knowledge about specific mental illnesses	
	β (95% CI )	*P*	β (95% CI )	*P*	β (95% CI )	*P*	β (95% CI )	*P*
Stage								
Baseline (ref)	−	−	−	−	−	−	−	−
Time 2	0.30 (0.26 to 0.34)	**<0.01**	0.20 (0.15 to 0.25)	**<0.01**	0.3 (0.26 to 0.34)	**<0.01**	0.20 (0.15 to 0.25)	**<0.01**
Time 3	0.31 (0.26 to 0.35)	**<0.01**	0.20 (0.16 to 0.25)	**<0.01**	0.31 (0.27 to 0.35)	**<0.01**	0.20 (0.16 to 0.25)	**<0.01**
Age	0^a^	0.33	0.00 (−0.00 to 0.00)	0.06	0.00 (−0.01 to 0.00)	0.33	0	0.16
Gender								
Male (ref)	−	−	−	−	−	−	−	−
Female	0.00 (−0.05 to 0.06)	0.87	−0.01 (−0.05 to 0.03)	0.54	−0.00 (−0.06 to 0.05)	0.93	−0.01 (−0.06 to 0.03)	0.06
Education								
None (ref)	−	−	−	−	−	−	−	−
Primary	0.01 (−0.10 to 0.11)	0.90	−0.04 (−0.12 to 0.05)	0.37	0.05 (−0.06 to 0.15)	0.40	−0.03 (−0.11 to 0.06)	0.55
Secondary	0.04 (−0.07 to 0.14)	0.50	−0.02 (−0.10 to 0.06)	0.64	0.09 (−0.02 to 0.19)	0.12	0.01 (−0.08 to 0.09)	0.87
Tertiary	0.15 (0.03 to 0.27)	**0**.**02**	0.02 (−0.08 to 0.11)	0.70	0.18 (0.05 to 0.31)	**0**.**01**	0.03 (−0.07 to 0.14)	0.51
Marital status								
Single (ref)	−	−	−	−	−	−	−	−
Married	−0.03 (−0.09 to 0.02)	0.24	0.03 (−0.02 to 0.07)	0.27	−0.01 (−0.08 to 0.07)	0.87	0.03 (−0.03 to 0.09)	0.34
Divorced/widowed/separated	0.08 (−0.01 to 0.17)	0.07	0.06 (−0.01 to 0.13)	0.10	0.12 (0.01 to 0.23)	**0**.**03**	0.07 (−0.02 to 0.15)	0.14
House tenure								
Rental (ref)	−	−	−	−	−	−	−	−
Owner	0.04 (−0.01 to 0.10)	0.12	−0.01 (−0.05 to 0.04)	0.75	0.06 (0.00 to 0.11)	**0**.**04**	−0.01 (−0.06 to 0.03)	0.56
Religious affiliation								
None (ref)	−	−	−	−	−	−	−	−
Islam	0.17 (0.00 to 0.34)	0.05	0.03 (−0.10 to 0.16)	0.67	0.20 (0.03 to 0.37)	**0**.**02**	0.02 (−0.12 to 0.15)	0.80
Christianity	0.17 (0.01 to 0.34)	**0**.**04**	0.05 (−0.08 to 0.18)	0.47	0.18 (0.02 to 0.34)	**0**.**03**	0.03 (−0.10 to 0.16)	0.63
Occupation								
Employed(ref)	−	−	−	−		−	−	−
Unemployed/student/other	0.04 (−0.02 to 0.09)	0.18	−0.01 (−0.05 to 0.03)	0.64	0.03 (−0.02 to 0.09)	0.25	−0.01 (−0.05 to 0.04)	0.75
Experience with mental illness								
No (ref)	−	−	−	−	−	−	−	−
Yes	0.05 (−0.03 to 0.13)	0.19	−0.00 (−0.06 to 0.06)	0.99	0.04 (−0.04 to −0.12)	0.35	−0.01 (−0.08 to 0.06)	0.79
Additional contact within the project								
No (ref)	−	−	−	−	−	−	−	−
Yes	0.08 (0.02 to 0.14)	**0**.**01**	0.03 (−0.01 to 0.08)	0.13	0.08 (0.02 to 0.14)	**0**.**02**	0.05 (−0.00 to 0.10)	0.07

ref, reference. Bold denotes *P*<0.05.

a. Zero denotes that estimates and both upper and lower confidence intervals were less than +/− 0.01.

In the multivariable analysis, general mental health-related knowledge scores improved immediately after watching the participatory videos (β = 0.30, 95% CI 0.26–0.34; *P* < 0.01) and remained higher than baseline scores 4 months later (β = 0.31, 95% CI 0.27–0.35; *P* < 0.01). Knowledge about specific mental illnesses also significantly improved at both follow-up time points (Table 2). Additional contact with the project (β = 0.08, 95% CI 0.02–0.14; *P* = 0.02) and tertiary level of education (β = 0.18, 95% CI 0.05–0.31; *P* = 0.01) remained significantly associated with higher general mental health-related knowledge. Additionally, people of Christian and Islamic faiths exhibited higher general mental health-related knowledge (Table 2). There were no significant sociodemographic correlates of scores on knowledge about specific mental illnesses.

### Changes in attitudes towards people with mental illnesses and sociodemographic correlates of attitude scores

All three domains of attitude improved at both time points after the intervention in both the univariable and multivariable analyses (Table 3). In the univariable analysis, compared with those with no formal education, having any form of education was significantly associated with a more liberal and sympathetic attitude but not with a more tolerant attitude. Compared with single people, people who were married (β = −0.21, 95% CI −0.36 to −0.07; *P* < 0.01) or who were divorced, widowed or separated (β = −0.27, 95% CI −0.48 to −0.06; *P* = 0.01) were significantly less liberal. Additional contact with the project through attending mass campaigns or other events was associated with a more tolerant attitude (β = 0.14, 95% CI 0.01–0.28; *P* = 0.04) but there were no associations with liberal or sympathetic attitudes. Older age was associated with a less liberal (β = −0.01, 95% CI −0.01 to 0.00; *P* < 0.01) but more sympathetic attitude (β = −0.01, 95% CI −0.01 to 0.00; *P* < 0.01). In the multivariable analysis, having any level of formal education was significantly associated with a more liberal and more sympathetic attitude. Age was associated with a more sympathetic attitude (β = 0.01, 95% CI 0.01–0.02; *P* < 0.01).

**Table 3:** Baseline and post-intervention attitude scores using the Community Attitudes to Mental Illness scale

Variable	Univariable analysis						Multivariable analysis					
	Factor 1 (Liberal attitude)		Factor 2 (Tolerant attitude)		Factor 3 (Sympathetic attitude)		Factor 1 (Liberal attitude)		Factor 2 (Tolerant attitude)		Factor 3 (Sympathetic attitude)	
	β (95% CI )	*P*	β (95% CI )	*P*	β (95% CI )	*P*	β (95% CI )	*P*	β (95% CI )	*P*	β (95% CI )	*P*
Stage												
Baseline (ref)	−	−	−	−	−	−	−	−	−	−	−	−
Time 2	0.61 (0.52 to 0.70)	**<0.01**	0.24 (0.14 to 0.35)	**<0.01**	0.44 (0.34 to 0.54)	**<0.01**	0.61 (0.52 to 0.70)	**<0.01**	0.24 (0.14 to 0.35)	**<0.01**	0.44 (0.34 to 0.54)	**<0.01**
Time 3	1.10 (1.00 to 1.17)	**<0.01**	0.72 (0.61 to 0.83)	**<0.01**	0.52 (0.42 to 0.62)	**<0.01**	1.08 (0.98 to 1.17)	**<0.01**	0.72 (0.61 to 0.83)	**<0.01**	0.52 (0.42 to 0.62)	**<0.01**
Age	−0.01 (−0.01 to 0.00)	**<0.01**	0.00 (−0.00 to 0.00)	0.95	0.01 (0.00 to 0.01)	**<0.01**	−0.01 (−0.01 to 0^a^)	0.05	0.00 (−0.00 to 0.01)	0.09	0.01 (0.01 to 0.02)	**<0.01**
Gender												
Male (ref)	−	−	−	−	−	−	−	−	−	−	−	−
Female	−0.10 (−0.23 to 0.02)	0.11	−0.01 (−0.13 to 0.12)	0.92	−0.05 (−0.20 to 0.09)	0.47	0.00 (−0.12 to 0.13)	0.98	0.03 (−0.10 to 0.16)	0.61	0.01 (−0.14 to 0.16)	0.90
Education												
None (ref)	−	−	−	−	−	−	−	−	−	−	−	−
Primary	0.48 (0.25 to 0.72)	**<0.01**	0.05 (−0.20 to 0.29)	0.72	0.48 (0.19 to 0.77)	**<0.01**	0.44 (0.20 to 0.69)	**<0.01**	0.10 (−0.15 to 0.35)	0.44	0.63 (0.34 to 0.92)	**<0.01**
Secondary	0.84 (0.61 to 1.08)	**<0.01**	0.13 (−0.11 to 0.38)	0.29	0.47 (0.18 to 0.76)	**<0.01**	0.79 (0.54 to 1.04)	**<0.01**	0.18 (−0.08 to 0.45)	0.17	0.66 (0.36 to 0.95)	**<0.01**
Tertiary	0.96 (0.69 to 1.23)	**<0.01**	0.28 (0.00 to 0.56)	0.05	0.77 (0.44 to 1.09)	**<0.01**	0.95 (0.65 to 1.25)	**<0.01**	0.30 (−0.01 to 0.61)	0.06	1.04 (0.69 to 1.40)	**<0.01**
Marital status												
Single (ref)	−	−	−	−	−	−	−	−	−	−	−	−
Married	−0.21 (−0.36 to −0.07)	**<0.01**	−0.14 (−0.28 to −0.00)	0.05	0.07 (−0.09 to 0.23)	0.41	−0.03 (−0.20 to 0.14)	0.75	−0.15 (−0.33 to 0.02)	0.09	−0.04 (−0.24 to 0.16)	0.72
Divorced/widowed/separated	−0.27 (−0.48 to −0.06)	**0**.**01**	−0.10 (−0.30 to 0.11)	0.36	0.19 (−0.06 to 0.43)	0.13	0.05 (−0.20 to 0.30)	0.70	−0.12 (−0.38 to 0.14)	0.36	0.10 (−0.20 to 0.39)	0.52
House tenure												
Rental (ref)	−	−	−	−	−	−	−	−	−	−	−	−
Owner	0.02 (−0.11 to 0.15)	0.75	0.01 (−0.12 to 0.13)	0.91	0.10 (−0.05 to 0.25)	0.18	0.13 (−0.00 to 0.26)	**0**.**05**	0.02 (−0.12 to 0.15)	0.78	0.06 (−0.09 to 0.21)	0.44
Religious affiliation												
None (ref)	−	−	−	−	−	−	−	−	−	−		−
Islam	0.44 (0.03 to 0.85)	0.04	0.13 ( to 0.27 to 0.53)	0.51	0.33 ( to 0.14 to 0.80)	0.17	0.30 ( to 0.09 to 0.68)	0.14	0.13 ( to 0.28 to 0.54)	0.53	0.12 ( to 0.34 to 0.58)	0.62
Christianity	0.37 (−0.03 to 0.77)	0.07	0.12 (−0.26 to 0.51)	0.53	0.36 (−0.09 to 0.82)	0.12	0.19 (−0.19 to 0.56)	0.33	0.09 (−0.31 to 0.48)	0.66	0.12 (−0.32 to 0.57)	0.59
Occupation												
Employed (ref)	−	−	−	−	−	−	−	−	−	−	−	−
Unemployed/student/other	0.08 (−0.21 to 0.05)	0.23	0.04 (−0.08 to 0.17)	0.50	0.03 (−0.18 to 0.12)	0.73	0.10 (−0.22 to 0.03)	0.13	0.00 (−0.13 to 0.13)	1.00	0.01 (−0.16 to 0.14)	0.93
Experience with mental illness												
No (ref)	−	−	−	−	−	−	−	−	−	−	−	−
Yes	0.14 (−0.33 to 0.05)	0.16	0.09 (−0.10 to 0.27)	0.35	0.17 (−0.39 to 0.04)	0.12	0.01 (−0.19 to 0.18)	0.96	0.07 (−0.13 to 0.27)	0.50	0.11 (−0.34 to 0.11)	0.33
Additional contact within the project												
No (ref)	−	−	−	−	−	−	−	−	−	−	−	−
Yes	0.02 (−0.16 to 0.12)	0.81	0.14 (0.01 to 0.28)	**0**.**04**	0.02 (−0.18 to 0.14)	0.84	0.09 (−0.24 to 0.06)	0.22	0.11 (−0.04 to 0.27)	0.15	0.01 (−0.17 to 0.18)	0.94

ref, reference. Bold denotes *P*<0.05.

a. Zero denotes that the upper confidence interval was less than 0.01.

### Changes in behaviour towards people with mental illnesses and sociodemographic correlates of behaviour scores

Compared with baseline, intended behaviour scores improved immediately after watching the participatory videos (β = −0.29, 95% CI 0.23–0.35; *P* < 0.01) and 4 months later (β = −0.37, 95% CI 0.31–0.43; *P* < 0.01), suggesting that the participatory videos may have been effective in lowering discriminatory behaviours among participants (Table 4). In both the univariable and multivariable analyses, secondary and tertiary levels of education were associated with higher intended behaviour scores, i.e. less discriminatory behaviour. In both models, compared with people with no religion, Christianity was associated with higher intended behaviour scores, i.e. Christians reported that they did not intend to engage in discriminatory behaviour.

**Table 4:** Baseline and post-intervention behaviour scores using the Reported and Intended Behaviours Scale

Variable	Univariable analysis		Multivariable analysis	
	β (95% CI )	*P*	β (95% CI )	*P*
Stage				
Baseline (ref)	−	−	−	−
Time 2	0.29 (0.23 to 0.35)	**<0.01**	0.29 (0.23 to 0.35)	**<0.01**
Time 3	0.37 (0.31 to 0.43)	**<0.01**	0.37 (0.31 to 0.43)	**<0.01**
Age, mean (s.d.)	−0.00 (−0.01 to 0.00)	0.08	−0.00 (−0.01 to 0.00)	0.36
Gender				
Male (ref)	−	−	−	−
Female	−0.00 (−0.09 to 0.09)	1.00	0.01 (−0.08 to 0.10)	0.89
Education				
None (ref)	−	−	−	−
Primary	0.16 (−0.01 to 0.33)	0.07	0.15 (−0.02 to 0.33)	0.09
Secondary	0.22 (0.04 to 0.39)	**0**.**01**	0.21 (0.02 to 0.39)	**0**.**03**
Tertiary	0.31 (0.11 to 0.50)	**<0.01**	0.26 (0.04 to 0.47)	**0**.**02**
Marital status				
Single (ref)	−	−	−	−
Married	−0.09 (−0.19 to 0.00)	0.06	−0.02 (−0.15 to 0.10)	0.70
Divorced/widowed/separated	−0.03 (−0.18 to 0.11)	0.68	0.08 (−0.10 to 0.26)	0.40
House tenure				
Rental (ref)	−	−	−	−
Owner	0.01 (−0.08 to 0.10)	0.78	0.06 (−0.03 to 0.16)	0.19
Religious affiliation				
None (ref)	−	−		−
Islam	0.26 (−0.02 to 0.54)	0.07	0.28 (0.00 to 0.57)	0.05
Christianity	0.29 (0.03 to 0.56)	**0**.**03**	0.30 (0.02 to 0.57)	**0**.**03**
Occupation				
Employed (ref)	−	−	−	−
Unemployed/student/other	−0.00 (−0.09 to 0.09)	0.94	−0.01 (−0.10 to 0.09)	0.88
Experience with mental illness				
No (ref)	−	−	−	−
Yes	0.05 (−0.08 to 0.18)	0.46	0.05 (−0.09 to 0.18)	0.49
Additional contact within the project				
No (ref)	−	−	−	−
Yes	0.10 (0.00 to 0.19)	0.04	0.08 (−0.03 to 0.18)	0.17

ref, reference. Bold denotes *P*<0.05.

### Interaction between intervention outcome and baseline characteristics in the knowledge domain

In the general mental health-related knowledge domain, significant differences were found in three baseline characteristics as follows: compared with those who rented houses, homeowners had lower levels of knowledge post-intervention (β = 0.05, 95% CI 0.00–0.09; *P* = 0.04); compared with those who were employed, those who were unemployed had higher levels of knowledge post-intervention (β = −0.06, 95% CI −0.10 to −0.01; *P* = 0.02); and compared with those who were not exposed to any additional components of the intervention, the exposed groups had higher levels of knowledge post-intervention (β = −0.09, 95% CI  = −0.14, −0.04; *P* < 0.01) (Table 5). In the knowledge about specific mental illnesses, significantly higher scores post-intervention were found only in those who were exposed to additional components of the intervention (β = −0.07, 95% CI −0.13 to −0.02; *P* = 0.01) (Table 5).

**Table 5:** Interaction analysis between sociodemographic variables and participatory video intervention in the knowledge domain

Variable	General mental health-related knowledge		Knowledge about specific mental illnesses	
	β (95% CI )	*P*	β (95% CI )	*P*
Age	0^a^	0.75	0^a^	0.59
Gender (male as reference category)				
Female	0.03 (−0.01 to 0.08)	0.15	0.02 (−0.03 to 0.07)	0.50
Education (None as reference group)				
Primary	−0.12 (−0.11 to 0.07)	0.67	−0.06 (−0.15 to 0.04)	0.25
Secondary	−0.12 (−0.11 to 0.07)	0.72	−0.06 (−0.16 to 0.04)	0.25
Tertiary	−0.10 (−0.20 to 0.00)	0.05	−0.08 (−0.19 to 0.03)	0.17
Marital status (Single as reference group)				
Married	0.04 (−0.01 to 0.09)	0.13	−0.02 (−0.08 to 0.03)	0.42
Divorced/widowed/separated	0.02 (−0.06 to 0.09)	0.61	0.04 (−0.05 to 0.12)	0.39
House tenure (Rental as reference group)				
House owner	0.05 (0.00 to 0.09)	**0**.**04**	−0.01 (−0.06 to 0.04)	0.72
Religious affiliation (No religion as reference group)				
Islam	−0.04 (−0.18 to 0.11)	0.61	0.11 (−0.05 to 0.27)	0.17
Christianity	−0.07 (−0.21 to 0.07)	0.31	0.04 (−0.11 to 0.20)	0.57
Occupation (Employed as reference group)				
Unemployed/student/other	−0.06 (−0.10 to 0.01)	**0**.**02**	0.01 (−0.04 to 0.06)	0.73
Experience with mental illness (No as reference group)				
Yes	−0.05 (−0.11 to 0.02)	0.18	−0.00 (−0.07 to 0.07)	0.98
Additional contact with other project components (No as reference group)				
Yes	−0.09 (−0.14, −0.04)	**<0.01**	−0.07 (−0.13 to −0.02)	**0**.**01**

Bold denotes *P*<0.05.

a. Zero denotes that estimates and both upper and lower confidence intervals were less than +/− 0.01.

### Interaction between intervention outcome and baseline characteristics in the attitude domain

In the liberal attitude domain, significant differences were found in five baseline characteristics as follows: with each unit increase in age, the intervention was less likely to improve participants’ attitude (β = 0.01, 95% CI 0.01–0.01; *P* < 0.01); compared with those who had no education, the intervention was more likely to improve scores among those with any level of formal education (Table 6); compared with those who were single, those who were presently married (β = 0.15, 95% CI 0.04–0.25; *P* = 0.01) or those who were divorced/widowed/separated (β = 0.33, 95% CI 0.17−0.48; *P* < 0.01) had significantly lower scores after the intervention; compared with those who rented houses, homeowners had lower levels of knowledge post-intervention and compared with those who were not exposed to any additional components of the intervention, the exposed groups were more liberal post-intervention (Table 6). In the tolerant attitude domain, significant differences were found in the following categories: marital status had a negative interaction with the domain, whereas additional contact had a positive interaction with the intervention outcome (Table 6). In the sympathetic attitude domain, experience with mental illness and additional contact with the project interacted with the intervention outcomes.

**Table 6:** Interaction analysis between sociodemographic variables and participatory videos intervention in the attitude domain

Variable	Liberal		Tolerant		Sympathetic	
	β (95% CI )	*P*	β (95% CI )	*P*	β (95% CI )	*P*
Age	0.01 (0.01 to 0.01)	**<0.01**	0.01 (0.00 to 0.01)	0.01	0.00 (−0.00 to 0.01)	0.12
Gender (male as reference category)						
Female	0.04 (−0.05 to 0.14)	0.35	−0.05 (−0.16 to 0.06)	0.38	−0.01 (−0.12 to 0.09)	0.82
Education (None as reference group)						
Primary	−0.23 (−0.42 to −0.05)	**0**.**01**	0.09 (−0.13 to 0.31)	0.44	0.05 (−0.16 to 0.26)	0.62
Secondary	−0.36 (−0.55 to −0.17)	**<0.01**	0.04 (−0.18 to 0.26)	0.71	0.06 (−0.15 to 0.27)	0.59
Tertiary	−0.46 (−0.67 to −0.25)	**<0.01**	−0.09 (−0.34 to 0.16)	0.47	−0.05 (−0.29 to 0.19)	0.68
Marital status (Single as reference group)						
Married	0.15 (0.04 to 0.25)	**0**.**01**	0.19 (0.06 to 0.31)	**<0.01**	0.11 (−0.00 to 0.23)	0.06
Divorced/widowed/separated	0.33 (0.17 to 0.48)	**<0.01**	0.24 (0.06 to 0.42)	**0**.**01**	0.09 (−0.08 to 0.27)	0.30
House tenure (Rental as reference group)						
House owner	0.18 (0.09 to 0.28)	**<0.01**	0.00 (−0.11 to 0.12)	0.96	0.10 (−0.00 to 0.21)	0.60
Religious affiliation (No religion as reference group) 0.22						
Islam	−0.24 (−0.54 to 0.07)	0.13	−0.22 (−0.58 to 0.13)	0.22	0.16 (−0.18 to 0.50)	0.35
Christianity	−0.22 (−0.51 to 0.09)	0.17	−0.20 (−0.55 to 0.14)	0.25	0.05 (−0.28 to 0.38)	0.78
Occupation (Employed as reference group)						
Unemployed/student/other	0.00 (−0.10 to 0.10)	0.99	−0.03 (−0.14 to 0.08)	0.61	−0.06 (−0.16 to 0.05)	0.31
Experience with mental illness (No as reference group)						
Yes	0.09 (−0.05 to 0.23)	0.23	−0.15 (−0.32 to 0.01)	0.07	−0.30 (−0.46, −0.15)	**<0.01**
Additional contact with other project components (No as reference group)						
Yes	−0.11 (−0.22 to −0.01)	**0**.**03**	−0.17 (−0.30 to −0.05)	**0**.**01**	−0.25 (−0.74 to −0.45)	**<0.01**

Bold denotes *P*<0.05.

### Interaction between intervention outcome and baseline characteristics in the behaviour domain

In the intended behaviour domain, level of education was the only baseline characteristic for which significant differences were found post-intervention (Table 7).

**Table 7:** Interaction analysis between sociodemographic variables and participatory videos intervention in the behaviour domain

Variable	β (95% CI )	*P*
Age	0.00 (−0.0 to 0.00)	0.07
Gender (male as reference category)		
Female	0.02 (−0.05 to 0.08)	0.62
Education (None as reference group)		
Primary	−0.20 (−0.33 to 0.07)	**<0.01**
Secondary	−0.20 (−0.32 to −0.07)	**<0.01**
Tertiary	−0.29 (−0.43 to −0.14)	**<0.01**
Marital status (Single as reference group)		
Married	0.02 (−0.05 to 0.09)	0.53
Divorced/widowed/separated	0.05 (−0.06 to 0.16)	0.37
House tenure (Rental as reference group)		
House owner	0.02 (−0.04 to 0.09)	0.54
Religious affiliation (No religion as reference group)		
Islam	−0.19 (−0.40 to 0.01)	0.07
Christianity	−0.20 (−0.40 to 0.00)	0.05
Occupation (Employed as reference group)		
Unemployed/student/other	−0.07 (−0.13 to −0.00)	0.05
Experience with mental illness (No as reference group)		
Yes	0.04 (−0.05 to 0.14)	0.38
Additional contact with other project components (No as reference group)		
Yes	−0.04 (−0.11 to 0.03)	0.23

Bold denotes *P*<0.05.

## Discussion

This study assessed the efficacy of a digital strategy utilising the method of participatory video to reduce public stigma against individuals with mental disorders in a resource-limited setting. Similar to a Canadian study^11^ employing participatory video for mental illness stigma reduction, we observed significant enhancement in knowledge, attitudes and intended behaviour after participatory video exercises. The effectiveness of our participatory video approach could be attributed to multiple interconnected components. First, involving individuals with lived experience as content creators is likely to have contributed positively to the outcomes. Content generated by those with lived experience of mental disorders tends to evoke emotions and is perceived as ‘real and relatable,’ ‘attention-grabbing,’ and ‘change-inducing’.^11^ Second, dissemination of the participatory videos in our study was conducted by individuals with lived experience, facilitating direct contact with the target audience. The efficacy of contact-based methods as stigma reduction strategies is well-documented.^28^ Third, the intervention targeted community opinion leaders, a proven strategy for enhancing mental health awareness and facilitating early intervention for common mental disorders such as depression.^29^

The successful implementation of the intervention hinged on the interplay between the technology (participatory video) and individuals embedded within a complex ecosystem of interacting factors. For instance, we capitalised on the administrative and social structures at our study site to engage community leaders as a target audience. However, infrastructure-related elements such as a consistent power supply and individual-level factors such as participants’ willingness to integrate the lessons into daily routines influenced the intervention outcomes. This implies that participatory video should not be seen merely as a product, but rather as a technology-enabled service with results contingent on addressing the needs of the target audience and adapting to the implementation environment. Thus, for widespread and sustainable adoption, future studies should customise such services to both audience needs and the implementation context.

Our findings of significant correlations between stigma scores and certain sociodemographic variables align with those of a study that conducted a digital stigma reduction campaign in Kenya and Ghana.^4^ However, these correlations were not uniform across all three stigma domains. This implies that when employing participatory video, it might be necessary to stratify the target population based on sociodemographic variables highly linked to the specific domain. For instance, our study revealed that practising any religion was associated with improved knowledge and behaviour outcomes, whereas a recent study in Kenya targeting youth aged 18–34 found better outcomes among those with no religion.^4^ This discrepancy might be attributed to the age-related mediation of religious affiliation,^30^ which is itself correlated with stigma.^31^ Consequently, for future studies aiming to enhance knowledge and intended behaviour in this context, age-based stratification of the target audience could be considered. Furthermore, the variability in sociodemographic correlates among the three stigma domains indicates that a singular intervention might not comprehensively address all aspects of stigma. This suggests that the application of the ‘what matters most’ principle in selecting the outcome variables of interest is important.^32–34^

The participatory video intervention was embedded within a broader community-level awareness campaign. Engaging in other aspects of the extensive campaign correlated with enhanced knowledge and improved attitudes, but not with positive intended behaviour; these outcomes were consistently observed in the interaction analysis. This indicates that further approaches are required for improved attitudes to translate into positive behaviour. When targeting opinion leaders, a more effective strategy for enhancing their intended behaviour might involve amalgamating mass campaigns with contact-based participatory video approaches. A future study including a control group is necessary to delve deeper into this observation. In contrast to other surveys, there was no correlation between lived experience of mental illness and overall stigma scores^4,35^ in our study. This may be due to lack of statistical power in our study to detect differences linked to lived experience.

### Study strengths and limitations

Our study contributes knowledge on the effectiveness of a participatory digital stigma reduction strategy in Africa, a region lacking extensive research on digital anti-stigma interventions.^5^ We employed globally validated stigma assessment tools,^4,36,37^ which were also validated in our study setting.^15^

However, our study does have limitations. The intervention comprised two key components: interactive educational participatory videos and in-person contact between the target audience and individuals with lived experience of mental illness. Although both strategies have shown lasting reductions in stigma,^38,39^ the exact mechanisms driving change in our study could not be specifically identified, warranting further qualitative exploration. Certain participants were exposed to other facets of the overall awareness campaign due to community residence, potentially influencing observed effects. Interaction effects between the participatory videos and mass community campaigns may have occurred, but owing to mass administration of the latter, such interactions could not be measured. Furthermore, absence of control groups not exposed to any interventions hindered the delineation of each component's relative contribution to the overall effect size. Despite sustained stigma reduction post-intervention for 4 months, the long-term impact remains unknown. Although the short follow-up periods minimised potential modification by other interventions, direct quantitative attribution of effects to the intervention was challenging. Lastly, social desirability bias due to face-to-face questionnaire administration, as seen in other studies,^40^ may have affected results.

### Implications

Although our study establishes evidence for the feasibility and efficacy of participatory approaches in reducing stigma, the diverse sociodemographic associations with stigma across the three domains suggest that future interventions may require targeted strategies for both components and desired outcomes. In this study, stigma levels were assessed using the biomedical model of illness understanding. To comprehensively address stigma, future research should explore it within alternative frameworks of mental health and illness, particularly in settings like ours, where culture significantly perpetuates stigma.^22^ Understanding the mechanisms underlying participatory video's efficacy as a stigma reduction strategy demands a collaborative effort involving mental health researchers, technology experts and behavioural scientists.

## Data Availability

All data are currently being curated to remove participant identifying information and will be made available on a publicly accessible website. Details of published data will be made available in future papers.
